# CAR-T Cell Therapy and Gut Microbiota Modulation in Multiple Sclerosis: Emerging Therapeutic Strategies and Current Limitations

**DOI:** 10.3390/cimb48090958

**Published:** 2026-09-19

**Authors:** Vitaly Chasov, Sabir Mukhametshin, Aygul Valiullina, Yulia Skibo, Maral Zhumabekova, Dinara Zharlyganova, Viktoriya Keyer, Aitolkyn Kydyrbayeva, Alexandr Shustov, Emil Bulatov

**Affiliations:** 1 Institute of Fundamental Medicine and Biology, Kazan Federal University, Kazan 420008, Russia; 2 National Center for Biotechnology, Korgalzhin hwy 13/5, Astana 010000, Kazakhstan; 3 Shemyakin-Ovchinnikov Institute of Bioorganic Chemistry, Russian Academy of Sciences, Moscow 117997, Russia

**Keywords:** multiple sclerosis, targeted therapies, CAR-T cell therapy, gut microbiota modulation

## Abstract

Multiple sclerosis (MS) is a chronic autoimmune inflammatory disease of the central nervous system (CNS) characterized by the destruction of the myelin sheath around nerve cells. The prevalence and serious consequences of MS highlight the shortcomings of existing treatments and the importance of developing innovative therapeutic approaches. Based on successful pilot studies in patients and an experimental autoimmune encephalomyelitis (EAE) mouse model, CAR-T cells offer a novel therapeutic mechanism by directly targeting and eliminating B cells, overcoming the shortcomings of antibody-mediated B cell depletion, which is unable to penetrate deep into the CNS. Recent discoveries have also revealed an important role for the gut microbiota in maintaining immune homeostasis. In a state of homeostasis, there is a symbiotic relationship between host factors and the microbiota that helps to maintain a healthy state. However, alterations in the composition and function of the gut microbiota, known as gut dysbiosis, can disrupt this homeostasis. The microbiota, through its metabolites, can influence not only internal processes in the gut but also the CNS in MS. Therapeutic interventions that help restore the balance of the gut microbiota may be a promising additional treatment to existing therapies to alleviate symptoms and promote remission in patients with MS.

## 1. Introduction

Multiple sclerosis (MS) is a disease caused by an imbalance of the immune system, due to which B and T cells become autoimmune and, after penetrating the blood–brain barrier, cause damage to the central nervous system (CNS) due to demyelination of the myelin sheath [1]. MS often develops as relapsing-remitting MS (RRMS), although a more acute form of the disease without remission also occurs, called secondary progressive MS (SPMS) [2]. There is also primary progressive MS (PPMS), which is diagnosed in about one in 10 patients, in which there is a gradual deterioration in the condition of patients without remission [2]. Although the etiology of MS is not fully understood, available data suggest that it may be associated with viral infections and environmental factors [3,4,5]. Genetic predisposition is another identified factor, with single-nucleotide polymorphism (SNP) mutations in human leukocyte antigen (HLA) genes being the most important identified risk locus for MS [6,7]. The frequency of SNPs in MS risk loci is known to be associated with exposure to environmental hazards such as cigarette smoke, heavy metals and other air pollutants, and low vitamin D levels [8,9,10,11,12,13].

Advances in recent years have led to the development of disease-modifying therapies (DMTs), which can slow the progression of the disease but do not provide a complete cure and have side effects [14]. Unfortunately, the benefits of DMT that have been observed in patients with RRMS are not typically seen in patients with SPMS and PPMS [15]. Even targeted therapy against immune cells, such as anti-CD20 B cell depleting antibodies (Abs), has some limitations in preventing long-term disability in progressive forms of MS [14].

As a result, there is growing interest in new targets and new strategies for the development of therapeutic drugs for MS. Chimeric antigen receptor T-cells (CAR-T cells) have an advantage over mAb therapy because they penetrate deeper into the CNS [16,17]. Recently, CAR-T therapy has been investigated for the treatment of patients with MS and in the EAE mouse model, showing promising results in terms of safety and efficacy [16,18].

In addition to environmental factors and genetic predisposition, the gut microbiota is now thought to play an important role in the development of MS and is being studied extensively [14]. Gut-associated microorganisms have been shown to be essential for maintaining the barrier function of the intestinal mucosa, as well as for the metabolism of many important nutrients in the host organism, playing regulatory and immunomodulatory roles [19]. Alterations in the composition of the microbiota, the characteristics of a healthy organism, can cause severe inflammatory responses in the host, including neurodegenerative consequences, and available data show significant differences in the gut microbiota between MS patients and healthy controls as well as in a mouse model of MS [14]. The idea of modulating the gut microbiota to restore its balance is attracting attention from researchers and may become a viable treatment for MS [20]. This review discusses the promising potential and drawbacks of a novel immunotherapeutic approach, CAR-T cell therapy, which may be used in clinical practice for the successful treatment of MS after appropriate clinical trials. We also summarize the relationship between gut microbiota dysbiosis and the pathophysiology of MS and promising therapeutic strategies targeting the gut microbiota to cure this disease.

## 2. Current Treatment of Multiple Sclerosis

**Figure 1:**
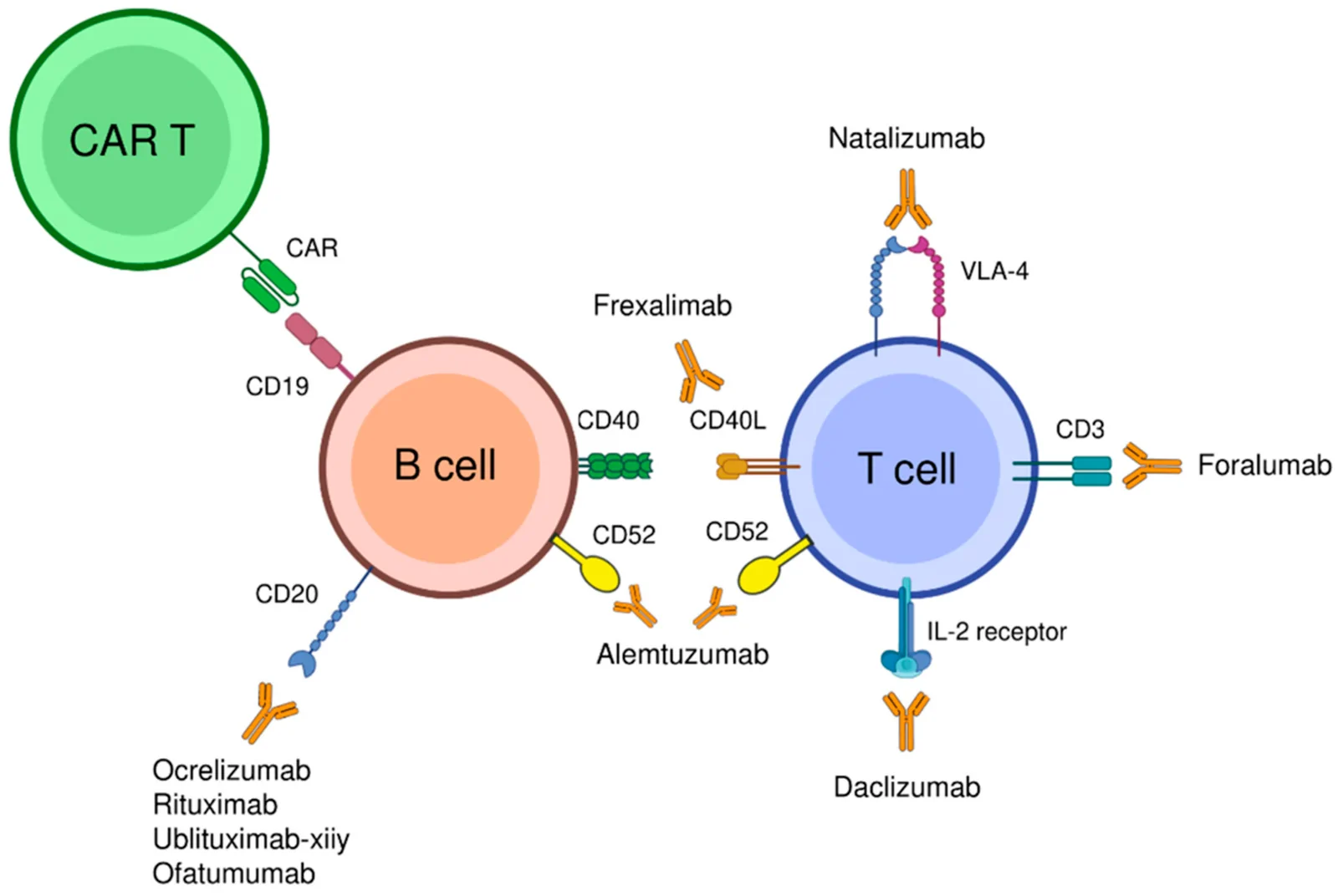
**Cellular targets of monoclonal antibodies and CAR-T cell therapy in multiple sclerosis (MS).** The schematic illustrates the principal immune cell targets and corresponding therapeutic agents used in the treatment of MS. Anti-CD20 monoclonal antibodies selectively deplete B cells, whereas antibodies targeting CD40/CD40L, CD52, VLA-4, CD3, and the IL-2 receptor modulate immune cell activation, migration, or proliferation. In contrast to monoclonal antibodies, anti-CD19 CAR-T cells are genetically engineered to recognize and eliminate CD19-positive B cells through direct cytotoxic activity. While several monoclonal antibodies are already approved for clinical use in MS and demonstrate high efficacy with acceptable safety profiles, preliminary clinical studies suggest that anti-CD19 CAR-T cell therapy represents a promising emerging therapeutic strategy for refractory and progressive forms of MS.

MS is currently divided into four different types, or disease courses, as defined by the International Advisory Committee on Clinical Trials of MS in 1996 [2]. They are: clinically isolated syndrome (CIS) that is first episode of neurologic symptoms, relapsing-remitting MS (RRMS), secondary progressive MS (SPMS), and primary progressive MS (PPMS) [2]. Although there is currently no complete cure for MS, research is underway to develop new and better DMTs, and DMTs can reduce the frequency and severity of MS attacks [21]. Among the current approaches to treat MS, targeted therapies such as mAbs are considered to be one of the safest options for long-term use (Figure 1, Table 1) [22].

### Monoclonal Antibodies

In MS, mAbs have emerged as some of the most advanced and effective therapeutics for specifically targeting autoimmune B and T cells to reduce inflammation and relapses in MS patients, including the first treatment for primary progressive MS, and are generally preferred due to their favorable side-effect profiles and dosing frequency (Figure 1, Table 1) [22]. Ocrelizumab is an anti-CD20 mAb that works by targeting, binding to and killing B cells, preventing them from entering the brain and spinal cord and thus reducing inflammation [23]. The FDA approved an intravenous infusion of ocrelizumab for the treatment of patients with both RRMS and PPMS in 2017, following successful phase III trials [24,25]. Research has shown that getting ocrelizumab infusions every 6 months can reduce relapse rates and slow the progress of disability for patients with PPMS and RRMS [25]. The data also showed that ocrelizumab was more effective than interferon beta-1a, which has been one of the main treatments for MS since the 1990s [25]. Currently phase IV clinical trial study the effects of early ocrelizumab treatment on B cells and T cells in patients with MS (NCT06495593).

Rituximab (Rituxan), another anti-CD20 mAb, is also used to treat MS. It demonstrated tolerability and efficacy in two randomized placebo-controlled phase II trials for RRMS and PPMS [26,27].

Ublituximab-xiiy (Briumvi) is a glycoengineered anti-CD20 mAb that was approved in the US in 2022 for the treatment of adults with relapsing forms of MS, including RRMS and SPMS [28]. It was approved by the FDA in 2022 [28]. A multicenter clinical trial of ublituximab for the treatment of recurrent forms of MS during phase II showed that ublituximab was safe and caused a significant decrease in the activity and frequency of relapses and depletion of B cells [29]. The phase III ULTIMATE I and II clinical trials (NCT03277261 and NCT03277248) also showed a statistically significant reduction in annualized relapse rate (ARR) and fewer brain lesions on magnetic resonance imaging (MRI) with ublituximab compared to teriflunomide [30].

Ofatumumab (Kesimpta) is a fully human anti-CD20 mAb that destroys B cells and can be self-administered by patients [31]. This medicine is approved in several countries around the world for the treatment of RRMS and SPMS, including FDA approval in 2020. The approval of ofatumumab was based on the results of several clinical trials. Phase III studies (ASCLEPIOS I and II) showed a decrease in the biomarker of neuroaxonal damage and the number of autoreactive B cells in serum [32]. MRI was used to study patients with recurrent forms of MS in the phase II of trials of ofatumumab, which revealed a significant decrease in the activity of new CNS lesions compared with baseline or compared with placebo [33,34]. The most common adverse effects following treatment with ofatumumab were headache, nasopharyngitis, upper respiratory tract infection and urinary tract infection [35]. It was concluded that ofatumumab is generally well tolerated and effective enough for the treatment of patients with RRMS [35].

Natalizumab is a mAb that used to treat patients with active RRMS, approved by FDA in 2004 [36]. The action of natalizumab is based on preventing the migration of autoimmune cells to the CNS due to binding to the alpha4-beta1 integrin receptor on leukocytes [36,37]. However, the use of natalizumab has been found to carry a high risk to induce progressive multifocal leukoencephalopathy (PML), an infection caused by human polyomavirus 2, also known as JC polyomavirus (JCV) [38]. PML after administration of natalizumab is a common complication among MS patients [39]. After reported mortality cases due to PML associated with natalizumab treatment, the FDA removed the drug from the market [40]. However, it was reintroduced in 2006 and patients should be closely monitored. Prescribers must be enrolled in specific safety programs, such as the TOUCH program in the USA.

Alemtuzumab is anti-CD52 mAb that has proven its effectiveness, as it selectively destroys autoimmune B and T lymphocytes, leading to long periods of remission, which allowed it to be approved for the treatment of RRMS [41,42]. However, there are frequent side effects associated with the use of alemtuzumab [43,44,45]. Approximately 38% of patients treated with alemtuzumab had thyroid-related adverse events [46]. Clinical trials have also documented nephropathy and thrombocytopenia in patients after alemtuzumab administration [47,48]. This limits the wider use of alemtuzumab.

Daclizumab is mAb that interacts with CD25, the alpha subunit of the IL–2 receptor, which inhibits T cell proliferation, and has been approved by the FDA and the EMA in 2016 for the treatment of relapsing forms of MS [49]. However, in 2018, it was removed from the market in the European Union due to serious side effects associated with liver and brain damage, and in the United States, its use is under strict control.

Frexalimab is a mAb whose activity is associated with blocking CD40/CD40L, one of the key pathways associated with activation of the immune system, leading to the formation of autoantibodies [50]. Clinical studies have shown the effectiveness of frexalimab in the treatment of patients with relapsing MS [50]. According to the results of MRI, more than 90% of patients had no Tesla 1 (Gd+ T1) lesions after 48 weeks of treatment, and during the study within 12 weeks, new lesions decreased by 89% compared with placebo [50]. Randomized, double-blind, placebo-controlled, parallel group study to determine the efficacy of frexalimab is in phase III now (NCT06141486).

Foralumab is a self-administered, fully human, intranasal anti-CD3 monoclonal antibody designed to bind to the T-cell receptor and attenuate inflammation by modulating T-cell function and suppressing effector functions in multiple immune cell subsets for the treatment of patients with MS [51]. Tiziana Life Sciences has received fast-track designation from the FDA for its intranasal foralumab for the treatment of non-active SPMS, a status granted with the aim of accelerating the development and review of potentially important drugs. Efficacy and safety of intranasal foralumab is currently being evaluated in a phase 2a, double-blind, randomized, placebo-controlled trial (NCT06292923).

## 3. CAR-T Cell Therapy

Although the antibodies described above, such as rituximab and ocrelizumab, are effective in preventing MS relapses, they cannot stop the disease from progressing [52]. This deficiency of antibodies can be explained by their inability to adequately affect the CNS, since they cannot overcome the protective barrier of the brain and spinal cord, where damage occurs [53]. Unlike B cell-targeting antibodies, CAR-T cells do not have these limitations, are autonomous and do not require macrophages, natural killer (NK) cells, or the complement system to function, and are able to achieve high levels of B cell depletion and penetrate deep into tissues of interest, such as the CNS [54]. CAR-T cell therapy involves redirected T-lymphocytes that are genetically engineered to bind to and recognize a specific antigen on a target cell [55]. To obtain CAR-T cells from a patient’s peripheral blood, T cells are isolated. The genome of these cells is modified by inserting the CAR gene. The cells are then cultured and used to treat patients [56]. The CAR sequence is not necessarily integrated into the genome, particularly when mRNA-based or other transient platforms are used [57]. Although CAR-T cell therapy was initially widespread in oncology, some progress has also been made in the treatment of systemic autoimmune diseases (SAIDs) [58,59]. Recently, CAR-T therapy has been investigated for the treatment of MS, where T-cells taken from patients are modified to target the B cells that attack the CNS in MS patients (Figure 1) [18].

This event was preceded by a study in a mouse model with MS-like experimental autoimmune encephalomyelitis (EAE) [16]. In this study, mice received pre-cyclophosphamide therapy [16].The study found that anti-CD19 CAR-T cell therapy in mice resulted in a reduction in autoreactive B cells in peripheral lymphoid tissue and the CNS [16].

Feasibility and safety profile of fully human anti-CD19 CAR-T cell therapy evaluated in two patients with SPMS and PPMS, respectively [18]. The presence and expansion of CAR-T cells was observed in the cerebrospinal fluid, which, together with the reduction in intrathecal antibody, suggests expansion-dependent effects of CAR-T cells on CD19+ target cells in the CNS [18]. At the same time, CAR-T cell administration resulted in a tolerable safety profile in both patients, with no clinical signs of immune effector cell-associated neurotoxicity [18].

It should be noted that the effectiveness of CAR-T cell therapy in significantly impacting the progression of MS and preventing disease relapses is currently a matter of debate (Table 2) [60,61]. It is suggested that CAR-T therapy is unable to effectively target the multifaceted components of MS and is similar to traditional mAbs to CD20 [61]. These mAbs are unable to selectively affect autoimmune B cells and also destroy regulatory populations of B cells [61]. It is emphasized that the mechanism by which CAR-T cell therapy can eliminate compartmentalized inflammation, which affects both B cells and microglia during MS progression is unclear [61]. This is an important point because CNS-compartmentalized autoreactive B cells have been shown to trigger CNS autoimmunity by interacting with pathogenic CD4+ T helper cells, requiring MHCII expression by B cells and GM-CSF production by T cells [62]. Opponents respond that, unlike mAbs, CAR-T cells actively penetrate tissues, including the CNS, and can provide much more potent local B cell depletion in patients with MS [60,63]. Although, the efficacy of anti-CD20 mAbs is not solely determined by CNS penetration, peripheral B cell depletion also influences antigen presentation, cytokine production, and B cell trafficking [64]. It is also important to note that the data on CAR-T cell migration into the CNS are preliminary and require further confirmation, and the presence of CAR-T cells in cerebrospinal fluid does not prove effective depletion of compartment-specific meningeal or parenchymal B cell populations [65]. However, anti-CD19 CAR-T cells may deplete a wider range of B-lineage cells than anti-CD20 mAbs. After treatment, the reconstituted B cells are mainly non-autoreactive, naive B cells, which induces a “reset” of the immune system [63]. In addition, CD20 is not expressed on plasmablasts or long-lived plasma cells, which continuously produce autoantibodies [64]. Another advantage of CAR-T therapy is that it usually only requires one treatment, whereas mAbs need to be given repeatedly due to their limited stability (Table 2) [66]. Considering new data on the mechanism of rapid progression in MS, this discussion is particularly relevant [67]. Using histological assays and spatial transcriptomics of brain autopsies from two groups of MS donors with slow and rapid disease progression, new data indicating that rapid disease progression is associated with myeloid cells located in the broad rim lesions was obtained [67]. These lesions exhibit elevated innate immune activity, which is linked to endoplasmic reticulum (ER) stress and the unfolded protein response (UPR) [67]. They also exhibit increased production of proinflammatory cytokines, including interleukin-8, as well as activation of receptor-interacting serine/threonine-protein kinase 1 (RIPK1)-associated pathways such as apoptosis and necroptosis [67]. These data suggest that broad-rim lesions could serve as a biomarker for rapid disease progression and as a new therapeutic target that is not addressed by current treatments.

The opposing views and open questions largely stem from the fact that CAR-T cell therapy for MS is still in its infancy. Future large-scale studies and clinical trials may clarify many of the controversies surrounding this therapeutic approach and define its boundaries and potential. If the trials are successful, CAR-T cell therapy could represent a significant breakthrough in MS treatment, offering patients long-term remission and improved quality of life. The approach, which involves producing CAR-T cells in vivo using CD8-targeted lipid nanoparticles (tLNPs) carrying anti-CD19 CAR mRNA, could help to achieve this goal by eliminating the need for laboratory cell manufacturing and ex vivo expansion [68]. Several clinical trials involving CAR-T cells in patients with MS have already begun. One of these is recruiting patients with relapsing or progressive forms of MS or refractory myasthenia gravis to evaluate the safety and tolerability of anti-CD19 CAR- T cells (NCT06220201, phase I). Another clinical study is evaluating the safety, tolerability, pharmacokinetics and efficacy profiles of LCAR-B38M, a CAR-T cell therapy, in patients with relapsed or refractory neurological autoimmune diseases, including MS (NCT06869278, phase I). Another study, which includes an autologous bispecific CAR-T therapy targeting CD20 and BCMA (C-CAR168), is recruiting patients with autoimmune diseases that do not respond to standard therapy, including MS (NCT06249438, phase I). Patients with non-relapsing and progressive MS (NCT06138132, phase I) and progressive MS (NCT06451159, phase I) are participating in a study of the anti-CD19 CAR-T cell therapy KYV-101. Finally, a clinical trial studying KYV-101 for the treatment of RRMS and SPMS is currently in phase II (NCT06384976).

Initial data show that CAR-T cell therapy is well tolerated by patients and has no significant side effects [18]. However, researchers note that, despite promising preclinical and clinical data, implementing CAR-T cell therapy for the treatment of MS into clinical practice is not an easy task [69]. It is essential to demonstrate the superiority of CAR-T cell therapy over currently available DMTs, including targeted therapies like mAbs, in terms of effectiveness, accessibility, and safety [70]. When comparing the therapeutic properties of mAbs and CAR-T cell therapy, in addition to the above, the following characteristics should be noted. Due to the fact that repeated antibody injections are often necessary, an insufficient dose of antibodies can lead to incomplete depletion and treatment failure [71]. On the other hand, antibody therapy allows for easier dosage control and adjustable treatment regimens based on the patient’s response, as opposed to CAR-T cells [71]. Therapeutic mAb-based therapy is more flexible and versatile than CAR-T cell therapy, because mAbs were not developed as personalized treatments for individual patients, and therefore they are more accessible and have a much lower cost [72]. In addition, CAR-T cell therapy requires a complex and time-consuming manufacturing process, which significantly limits its widespread availability [73]. Therefore, compared to CAR-T cell therapy, antibodies are currently much more widely applicable due to their simplicity of application, reproducibility of results, and scalability for mass production (Table 2) [74]. The possible local immune effector cell-associated toxicity syndrome (LICATS), which occurs when anti-CD19 CAR-T cells penetrate deep tissue, should also be considered [75,76]. However, the profound consequences of this phenomenon have not yet been described. In addition, when using CAR-T cell therapy for MS, potential side effects such as cytokine release syndrome (CRS) and immune effector cell-associated neurotoxicity syndrome (ICANS) may occur because the pathogenic cells are located in the CNS, which is a microenvironment rich in microglia and prone to inflammation [70]. Pre-existing CNS inflammation and altered blood–brain barrier permeability in MS patients may increase the risk of neurotoxicity during CAR-T cell therapy, compared to patients with hematological malignancies, due to pre-activated local immune cells and easy entry of therapeutic cells and cytokines into the brain [70]. Risk minimization strategies may include the use of transient CAR expression systems based on mRNA or non-viral platforms, the incorporation of antibody-based or genetic safety switches, and the use of CAR-engineered regulatory T cells (CAR-Tregs) and NK (CAR-NKT) cells [70]. Prolonged cytopenia, infections, and hypogammaglobulinemia may also occur, which can be life-threatening and require continuous monitoring and treatment [77].

**Figure 2:**
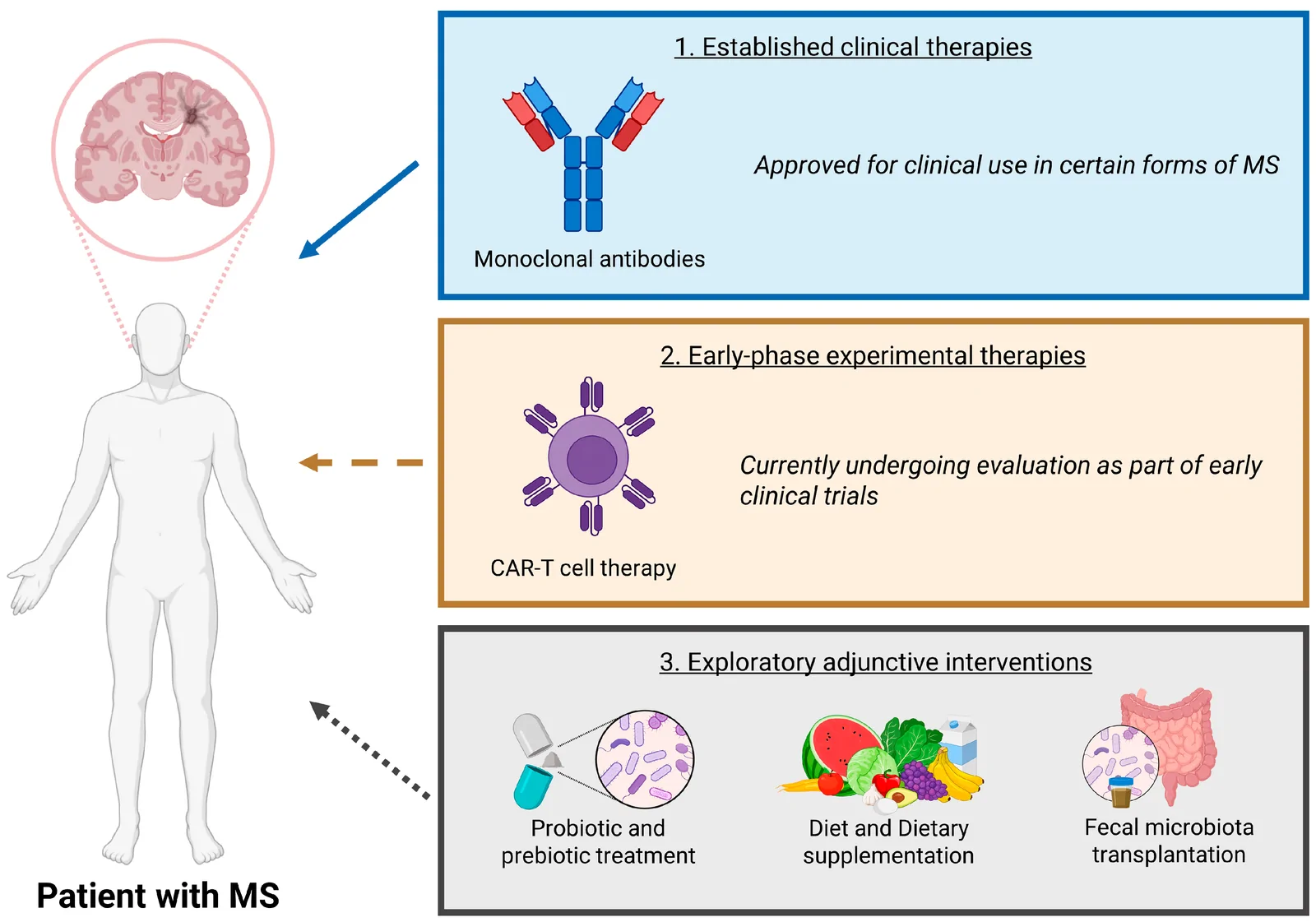
**Overview of established and emerging therapeutic strategies for multiple sclerosis (MS).** The schematic summarizes current and prospective approaches for the treatment of MS. The numbers indicate levels of clinical validation, including monoclonal antibodies that are established disease-modifying therapies with proven clinical efficacy (1); anti-CD19 CAR-T cell therapy, which represents a promising immunotherapeutic approach that may provide deeper and more sustained depletion of pathogenic B cells, although its long-term safety and superiority over existing mAbs remain to be confirmed in large clinical trials (2); and exploratory adjunctive interventions targeting the gut microbiota, such as probiotic and prebiotic treatment, dietary interventions and supplementation, and fecal microbiota transplantation, aim to restore gut microbial homeostasis and modulate immune responses (3). While these approaches have shown encouraging preclinical and early clinical results, further studies are required to establish their efficacy, safety, standardization, and applicability in routine clinical practice.

Since even CAR-T therapy currently has certain limitations, such as the complexity of the procedure, high cost, and potential side effects, it is becoming clear that the development of new therapeutics for MS treatment associated with reduced disease relapse, brain damage and systemic side effects is an urgent task (Table 2). One such alternative method is discussed below (Figure 2).

## 4. Gut Microbiota in the Pathogenesis of Multiple Sclerosis

### 4.1. Links Between the Gut Microbiota and Multiple Sclerosis

The amount and diversity of gut microbiota vary between individuals and depends on many factors, but some phyla were found to be the most dominant, including Firmicutes, Actinobacteria, Proteobacteria and Bacteroidetes [78,79]. To maintain the homeostasis characteristic of a healthy organism, a physical barrier is required to prevent external microorganisms from penetrating the intestinal wall [80]. This barrier is provided by mucus, the secretion of enzymes, antimicrobial proteins, and immunological factors such as IgA that control bacterial colonization [81]. When discussing the relationship between MS development and gut microbiota composition, it should be noted that there is no established cause-and-effect relationship between these phenomena, and we can only talk about an association between them. Differences in microbiota between patients with MS and healthy individuals can also be due to diet, geography, age, smoking, constipation, disability, disease activity, past disease-modifying therapies, corticosteroids, antibiotics and testing methods [19,82,83,84].

To assess the significance of the microbiota in MS pathogenesis, a unique experiment was conducted to examine the fecal microbiota of monozygotic twin pairs discordant for MS [85]. This allowed many concomitant genetic and environmental factors to be minimized [85]. In this study, microorganisms obtained from different regions of the intestine were transferred to germ-free (GF) mice [85]. It was found that the ileal microbiota obtained from twins with MS caused EAE at a significantly higher rate than similar material from healthy twin donors [85]. The experiment identified two species—*Eisenbergiella taiyi* and *Lachnoclostridium*—belonging to the *Lachnospiraceae* family, which are capable of causing disease in mice, indicating a potential role in the development of MS in humans [85].

The composition of the gut microbiota in stool samples from patients with newly diagnosed MS was evaluated using sequencing analysis at baseline and after B cell depletion by anti-CD20 mAb within six months [86]. The study showed that B cell depletion was associated with changes in the abundance and prevalence of certain organisms [86]. For example, the relative abundance of *Mogibacterium diversum*, *Odoribacter splanchnicus*, *Pseudoflavonifractor* and *Desulfovibrio* increased after six months of B cell depletion, while *Akkermansia muciniphila*, *Bifidobacterium bifidum*, *Blautia obeum* and *Gemella* displayed immune coating patterns that were more similar to those observed in the control group [86]. These data indicate not only a close relationship between gut microbiota and disease, but also that immune perturbation due to B cell depletion influences gut microbiota dynamics [86].

Recent study analyzing the gut microbiome of 576 patients with MS and household healthy controls (1152 total subjects) revealed significant increases in the abundance of *Ruthenibacterium lactatiformans*, *Hungatella hathewayi* and *Eisenbergiella tayi*, and decreases in *F. prausnitzii* and *Blautia* species, as the disease progresses [87]. It is important to note the reduction in *F. prausnitzii* in MS, which produces short chain fatty acid (SCFA), a metabolite of great importance in maintaining immune homeostasis in MS as it reduces the production of proinflammatory molecules such as iNOS, TNF-α, MCP-1, IL-12 and IL-6 [87]. Levels of SCFA-producing Bacteroidaceae and *Clostridium* clusters XIVa and IV of the phylum Firmicutes are also reduced in MS, according to bacterial 16S ribosomal RNA (rRNA) gene sequencing analysis [88]. Clostridia strains were found to be involved in the maintenance of Treg cells required for immune tolerance [89].

Several independent studies have shown that *Prevotella* is inversely correlated with MS severity, suggesting that this genus may have an anti-inflammatory or other protective role in MS [90,91,92]. In addition, the number of intestinal Th17 cells, which induce brain autoimmunity, was shown to be inversely correlated with the relative abundance of *Prevotella* species in the human small intestine [93]. At the same time, after treatment with IFN β-1b, the abundance of *Prevotella copri* in MS patients was increased to the same level as in healthy controls [92]. Following use of this drug, a trend towards a healthy control was also observed for other gut bacterial species with altered abundance in MS: Firmicutes, Actinobacteria and Lentisphaerae [92].

The next study sequenced the microbiota of healthy controls and RRMS and progressive MS patients [94]. The increased *Clostridium bolteae*, *Ruthenibacterium lactatiformans* and *Akkermansia* and decreased *Blautia wexlerae*, *Dorea formicigenerans* and Erysipelotrichaceae CCMM were found in both progressive MS and RRMS [94]. Increased Enterobacteriaceae and *Clostridium* g24 FCEY and decreased *Blautia* and *Agathobaculum* were found to be unique to progressive MS [94]. Several *Clostridium* species were also associated with higher Expanded Disability Status Scale (EDSS) and fatigue scores [94]. *Akkermansia* isolated from MS patients has been reported to ameliorate EAE, suggesting a beneficial role in MS [94]. Synthetic miR-30d administered orally was also found to increase the abundance of *Akkermansia* in the gut, which in turn increases Tregs and thus ameliorates EAE [95]. Mendelian randomisation analyses using three large genome-wide association studies (GWASs) of the gut microbiome confirmed a strong protective role for both *Akkermansia municiphila* and *Prevotella copri* in MS susceptibility [96]. The same analysis showed that the relative abundance of *Ruminococcus torques*, Actinobacteria, *Bifidobacterium adolescentis*, *Bifidobacterium bifidum* and *Lactobacillus* increased the risk of MS [96]. However, the role of *Akkermansia muciniphila* in the pathogenesis of MS requires further investigation, as it has been found that immunoglobulin G (IgG) responses against *A. muciniphila* in the cerebrospinal fluid of MS patients correlate with disease activity and CNS damage [97].

It should be noted that intestinal bacteria largely modulate immunity through their metabolites. Deoxycholic acid (DCA) and lithocholic acid (LCA) are immune-regulatory bile acid (BA) metabolites produced by beneficial bacteria. They have been shown to decrease in patients with MS who have an increased percentage of Th17 cells [98]. Administration of DCA/LCA in the EAE model prevents disease by suppressing the differentiation of Th17 and effector myelin-reactive T cells [98]. The following study found that changes in serum BAs in patients with MS compared to controls are associated with the risk of disability worsening, thus confirming the importance of this bacterial metabolite in maintaining immune system balance [99]. A large, multi-method study revealed that the transition from RRMS to progressive MS is linked to alterations in gut microbiota and metabolites [100]. Beneficial associations with *Blautia*, *Eubacterium* and *Akkermansia*, and negative associations with *Alistipes* and *Bacteroides*, were found consistently across several measures of disease progression [100]. In samples from patients with progressive MS, serum levels of palmitoleate and p-cresol sulphate increased, while ursodeoxycholate and isoursodeoxycholate decreased [100]. In fecal samples, the levels of beneficial metabolites such as nicotinate (vitamin B3), nicotinate ribonucleoside and protoporphyrin IX, as well as sphingolipids including sphingosine and sphingomyelin, were lower in patients with progressive MS [100].

### 4.2. Therapeutic Strategies for Targeting Gut Microbiota in the Treatment of Patients with MS

Considering the well-established link between gut dysbiosis and MS, a number of approaches have been proposed to improve the composition of gut microbiota, which are often seen as complementary therapies. These methods can be suggested as a valuable addition to existing therapeutic approaches, which have proven to be promising (Figure 2).

#### 4.2.1. Probiotic and Prebiotic Treatment

One way to influence the composition of bacteria in the gut is using probiotics, which are live beneficial microorganisms [101]. Among the most used probiotics are *Lactobacillus* and *Bifidobacterium*, both of which produce SCFAs, and have been studied in patients with MS and EAE models [102]. In a mouse model of MS, administration of a polyprobiotic consisting of *Lactobacillus casei*, *Lactobacillus acidophilus*, *Lactobacillus reuteri*, *Bifidobacterium bifidum* and *Streptococcus thermophilus* resulted in an increase in Treg cells and increased secretion of the anti-inflammatory cytokine IL-10 [103]. Administration of *Lactobacillus plantarum* plus *Bifidobacterium animalis* as a probiotic was also shown to reduce neuroinflammation and increase Treg cells in the lymph nodes and spleen [104]. Another study found that *Lactobacillus reuteri* treatment in an EAE model restored the diversity of the gut microbiome and reduced Th1 and Th17 cells and their corresponding cytokines [105]. A mixture of probiotics, consisting of *Lactobacillus* and *Bifidobacterium*, was also found to have an anti-inflammatory effect in MS patients, resulting in a reduction in the autoimmune activity of immune system cells [106].

In addition to the above, other strains have caught the attention of researchers. After introducing *Bacillus coagulans* into mice with EAE, it was found that there was also a reduction in proinflammatory cytokines [107]. This strain is known to exert an anti-inflammatory effect by suppressing the expression of the IDO enzyme, resulting in reduced degradation of tryptophan via the kynurenine pathway, which in turn increases the availability of tryptophan for serotonin synthesis [107]. This suggests the ability to selectively regulate metabolic pathways, which is another avenue for probiotic therapy in MS [108]. It has been reported that it is important to consider the environment when treating probiotics, as the same probiotic strain may act differently when given in combination with different probiotic bacteria [109].

The beneficial properties of some strains related to their production of outer membrane vesicles (OMV) are also discussed [108]. In addition to direct interactions, OMVs are the part of an alternative horizontal communication system and act as the messengers between bacteria and their host [110,111]. OMVs are thought to have beneficial effects in MS therapy [108]. For example, OMVs produced by *Bacteroides fragilis* are recognized by DCs via TLR2, leading to an increase in the production of anti-inflammatory cytokines and the number of Tregs [112]. *Akkermansia muciniphila* has also been found to produce OMVs, which promote the secretion of serotonin in the hippocampus and colon [113]. Taken together, these results indicate the important role of probiotics in restoring the balance of the gut microbiota and the potential for their use in the treatment of MS following appropriate clinical trials.

Prebiotics are non-living food components that are not digested or absorbed in the upper gastrointestinal tract, including fiber, but stimulate the growth and activity of intestinal bacteria [114]. Prebiotics are always present naturally in the diet but can also be added intentionally. It is known that adding prebiotics such as inulin, fructooligosaccharides and lactosucrose, the main energy sources for bacteria, to the diet increases the production of SCFAs and the regulation of intestinal mucins [115,116]. Prebiotics can be used in combination with probiotics to increase the survival and functionality of probiotic bacteria [117,118]. The specific mixtures of prebiotics and probiotics intended to provide health benefits to the host by stabilizing and supporting gut microbiota are called synbiotics. This synergistic approach has great potential to correct gut microbiota dysbiosis and normalize immune system functions in the treatment of MS.

#### 4.2.2. Diet and Dietary Supplementation

The imbalance of the intestinal barrier, or the “leaky gut” phenomenon, observed in MS patients is one of the mechanisms through which the gut microbiota may influence MS pathology [119,120]. Food components are known to have either anti-inflammatory or proinflammatory effects in MS pathogenesis and influence the human metabolism, the composition of gut microbiota and intestinal barrier function [121,122,123]. SCFAs produced by beneficial bacteria, pectin obtained through diet and mucin secreted by goblet cells all support healthy gut barrier function [124,125,126]. It is thought that an elevated level of the pectin-degrading bacterium *Monoglobus pectinilyticus* in patients with MS is associated with the “leaky gut” phenomenon [86]. High-calorie, animal-fat-rich diets are claimed to contribute to an imbalance of the gut microbiota [121,122,123]. Subsequently, this can lead to a disruption of the intestinal barrier function and penetration of foreign antigens through the blood–brain barrier, causing neuroinflammation [121,122,123]. In contrast, a balanced diet, especially the Mediterranean diet, which is rich in fiber and monounsaturated fatty acids, can help maintain a healthy intestinal barrier [121,122,123]. The beneficial effects of vitamin D are also well known, and it is considered one of the most promising dietary molecules for the treatment of autoimmune diseases, including MS [127]. The study investigated the effect of vitamin D and glatiramer acetate on the intestinal microbiota composition in MS patients compared to healthy controls [128]. The study found that patients with MS who took vitamin D supplements experienced an increase in the numbers of bacteria from the genera *Faecalibacterium*, *Akkermansia*, and *Coprococcus*, compared to the control group [128].

Lifestyle behaviors could be beneficial for patients with MS, including personalized dietary interventions in accordance with the principles of a healthy diet described above. Human studies have also demonstrated that home-based exercise training has a positive effect on gut bacteria, leading to improvements in some symptoms of MS [129]. In addition, further research has reported the benefits of lifelong physical activity on brain health, including reduced lesion volume and bacterial count in MS [130].

Studies have shown that adding bacterial metabolites to the diet also has a therapeutic effect in the treatment of MS [131,132]. This is particularly true of BAs. BAs are involved in the digestion and absorption of fats and are an important product of cholesterol metabolism [131]. There is a wide variety of BAs in the intestine due to the conversion of primary BAs into secondary BAs by bacteria. These secondary BAs can also be metabolized further [133]. Among BAs, primary BAs such as cholic acid and chenodeoxycholic acid, and secondary BAs, such as lithocholic acid and ursodeoxycholic acid (UDCA), enter the bloodstream from the intestine and are involved in various metabolic processes [133,134]. Available data suggest that the gut microbiota, including the genera *Bacteroides*, *Prevotella* and *Ruminococcus*, align with differences in microbial metabolic capacity for BA transformation [135]. Communities dominated by *Bacteroides* typically display high deconjugation capacity, but limited secondary BA production [136]. In contrast, communities enriched with *Prevotella* emphasize carbohydrate fermentation [136]. Enterotypes associated with *Ruminococcus* harbor extensive secondary BA-producing machinery, generating metabolites that have potent effects on the farnesoid X receptor (FXR) and Takeda G protein-coupled receptor 5 (TGR5) [137]. These differences may contribute to the variability in metabolic responses to dietary interventions that is observed in 30–60% of individuals, which are typically designed based on average data from populations [138]. BAs have been found to reduce the expression of MHC-II and CD80 by interacting with monocytes and microglia through a special receptor, GPBAR1 [139]. Tauroursodeoxycholic acid (TUDCA), a more water-soluble derivative of UDCA, has been found to promote anti-inflammatory signaling in microglia, and TUDCA supplementation at a dose of 500 milligrams per kilogram, administered orally, prevents the polarization of microglia and astrocytes into neurotoxic phenotypes and reduces the pathological processes in EAE model of MS [140]. In addition, obeticholic acid, a synthetic BA agonist of the farnesoid X receptor has been shown to ameliorate MS, suppressing lymphocyte activation and proinflammatory cytokine production in EAE model [141]. A double-blind, placebo-controlled trial involving 47 patients with PMS revealed that TUDCA supplementation was safe and well tolerated [131]. It was found that after TUDCA supplementation memory CD4+ and Th1/17 cells decreased, while CD4+ naive cells increased [131]. The composition and function of the gut microbiota changed compared to the placebo group, without any differences in adverse events between the groups [131].

SCFAs are the main products of the fermentation of carbohydrates, such as dietary fiber [138]. Acetate, a simple SCFA that consists of two carbon atoms, is one of the common end products of glycolysis in various commensal microorganisms such as *Bacteroides*, *Bifidobacterium*, *Blautia*, and *Marvinbryantia* [142]. Propionate with three carbon atoms is mainly produced through the succinate, acrylate, and propanediol pathways within specific bacteria of the *Lachnospiraceae* family and *Negativicutes* class, as well as *Bacteroides* and *Akkermansia* [143]. Butyrate, another SCFA with four carbon atoms and known as a major energy source for colon epithelial cells, is produced by specific bacteria belonging to Clostridia such as *Faecalibacterium*, *Eubacterium*, *Roseburia*, and *Ruminococcus* [144]. SCFAs have also shown promise as dietary supplements [132,145]. SCFA supplementation has been shown to reduce the clinical severity of MS and inflammation in EAE models [145]. And SCFA supplementation in the drinking water of GF mice has been shown to induce anti-inflammatory processes in microglia [146]. Propionic acid in particular appears to be promising, as treatment with propionic acid was associated with a reduction in Th17 cells and an increase in the number and regulatory functions of Tregs [147].

The researchers also found that taking N-acetylglucosamine as a dietary supplement can reduce inflammation and may help repair myelin in patients with MS [148].

These results suggest that bacterial metabolites have the potential to influence the progression of the disease and regulate the immune response, which could be significant for the treatment of MS. However, it should be noted that the optimal duration and dosage of BAs and SCFAs administration in patients with MS have not yet been established, and therefore, the problem of achieving consistent treatment outcomes has not been resolved [149]. Another limitation is the complexity of the gut microbiota, and the therapeutic effect is largely dependent on the composition of the gut microbiota of a particular patient [149].

#### 4.2.3. Fecal Microbiota Transplantation

One of the most promising ways to modulate the composition of the gut microbiota for therapeutic effects in MS is through fecal microbiota transplantation (FMT). This procedure involves the administration of fecal bacteria from a healthy donor into the intestinal tract of a recipient to directly change the gut microbial composition [150]. FMT has been shown to be effective and safe in the treatment of other autoimmune diseases such as SLE and RA [151,152]. It is not surprising that the therapeutic potential of this approach has also been the subject of interest in the field of MS [153]. In a mouse model of MS, FMT has been shown to lead to the formation of a health-promoting bacterial composition, reduce demyelination and axonal loss, and confer protection to the blood–brain barrier [154]. Nine patients with relapsing MS were then recruited for a randomized controlled pilot trial of FMT, and now it is in phase II (NCT03183869) [155]. This study assessed gut microbiota composition, gut permeability and safety of the FMT procedure [155]. The results showed significant, donor-specific, beneficial changes in the patients’ gut microbiota and an improvement in elevated small intestinal permeability [155]. At the same time, FMT was safe and well tolerated in this patient population [155].

There are also reports of other clinical trials studying FMT in patients with MS. Two phase I trials investigating the oral (NCT04096443) or colonoscopic (NCT03594487) administration of FMT have been initiated. A phase I trial is also underway to study whether FMT can improve the outcome of autologous hematopoietic stem cell transplantation in patients with MS (NCT04203017). A placebo-controlled, double-blind, randomized trial of FMT for relapsing MS patients is in phase II (NCT04150549). Overall, these results are promising. However, further studies with longer follow-up periods and larger sample sizes are needed to determine the feasibility of using FMT in the clinical treatment of MS.

For widespread implementation of FMT, protocols should be standardized to eliminate variability in results and increase reproducibility [156]. Careful screening of donors is also required to eliminate the risk of transmission of pathogens to patients [157]. It should be noted that existing data on FMT use in MS is based on small pilot studies, which provide insufficient information about neurological efficacy and long-term safety. Risks of pathogen transmission, multidrug resistance, and poorly understood microbiome characteristics need to be acknowledged. Therefore, more rigorous studies are needed to confirm safety and efficacy.

#### 4.2.4. Gut Microbiota and CAR-T Cell Therapy Integration

It is important to note that the potential clinical combination of CAR-T therapy and microbiota modulation is possible, but requires careful consideration. Available data point to the integration of gut microbiota monitoring into CAR-T cell preconditioning protocols, because the state of the microbiota may be associated with both the efficacy and toxicity profile of CAR-T cell therapy [158]. Gut microbiota therapy interventions, such as probiotics and FMT, may carry additional infectious risks for patients undergoing lymphodepletion or experiencing prolonged B cell aplasia, hypogammaglobulinemia, or cytopenia, and they are required validation in prospective interventional trials in the context of CAR-T cell therapy [158]. At the same time, greater gut microbiota diversity and the abundance of specific butyrate-producing bacteria were found to be associated with more favorable and safer responses to CAR-T therapy in clinical studies [159,160]. In turn, CAR-T cell therapy may also have a negative impact on the gut microbiota. However, the primary driver of gut dysbiosis during CAR-T treatment is often heavy use of broad-spectrum antibiotics and conditioning chemotherapy, rather than CAR-T cells themselves [158]. As a result, dysbiosis may reduce the effectiveness of CAR-T therapy backward. Observations from CAR-T cohorts have revealed that exposure to antibiotics before CAR-T infusion, which disrupts the gut microbial composition, is associated with higher risks of severe toxicity and reduced treatment efficacy [161]. The individualized nature of the human microbiome means that there is no one-size-fits-all product to maintain a healthy composition of gut bacteria during CAR-T therapy. We suggest that managing dysbiosis of the gut microbiome during CAR-T cell therapy may involve the use of smart antibiotic choices, dietary changes, and restorative treatments, such as carefully selected and specially designed probiotics.

## 5. Conclusions and Further Perspectives

MS is a chronic autoimmune disease of the CNS that is a major public health problem and patients with MS experience significant neurological impairment. Despite significant advances in immunosuppressive and immunomodulatory DMTs, which are currently widely used in clinical practice and can effectively delay progression of RRMS during exacerbations, most patients still progress to SPMS, which is largely unresponsive to DMTs. Effective treatment is also not yet available for patients with PPMS. In addition, DMTs often have a number of side effects that need to be considered when using them for long-term treatment. As a result, new options for treating MS have emerged, such as immunotherapy. Unlike B cell-targeting Abs, which are unable to adequately access the CNS, anti-CD19 CAR-T cells act autonomously, penetrating deep into tissues and effectively depleting B cells, particularly in the CNS. Although the deep penetration of CAR-T cells into the CNS is still being investigated, further research is required to confirm this. Early results from the use of CAR-T cells in MS are promising, and this therapy has the potential to treat patients as their disease progresses. However, even CAR-T cell therapy have limitations related to the complexity of the procedure and high cost. Moreover, the efficacy of anti-CD19 CAR-T cell therapy and its ability to selectively destroy B cells, including the compartmentalized CNS structures compared to anti-CD20 mAbs is still debated. Determining the safety, efficacy and applicability of CAR-T cell therapy in further clinical trials are important goals.

For these reasons, there is a growing need to develop alternative and complementary treatments. In recent years, researchers have become interested in the possibility of using dysbiosis correction of the intestinal microbiota to improve the condition of patients with MS. This possibility is based on the well-established correlation between dysbiosis of the gut microbiota and the functioning of the immune system. This affects the autoimmune response in MS. From this perspective, targeting the gut microbiota is the approach that optimizes the use of resources, as it does not require expensive therapies, but only uses the body’s own resources. In addition to their cost-effectiveness, microbiota-targeted treatments have a general strengthening and healing effect that goes beyond a specific disease. They are also a good way to prevent disease. However, it should be noted that there are differences in the composition and relative proportions of various microorganisms associated with MS among studies conducted by different researchers using different methods. This must be considered, which complicates the development of methods for correcting the intestinal microbiota and introducing them into clinical practice for treating autoimmune diseases, including MS. Further research is needed to clarify this issue. Basically, the balance of the gut microbiota can be controlled by probiotic and prebiotic treatments, special diets and supplements, and FMT. Despite the proven therapeutic effect, the use of probiotics has significant limitations. For example, the effect of probiotics is difficult to predict in the long term in different populations, as their effect is highly dependent on the diet and environment. In addition, the dosage and duration of probiotic treatment have not been specified. Diet is considered an important factor in managing MS, as it can affect the composition of the gut microbiome, which in turn affects immune balance and intestinal barrier function through the metabolites it produces. Although FMT appears to be relatively safe and effective in treating a wide range of diseases, including MS, according to pilot studies, its safety and efficacy in usual clinical settings remain unknown. More data is required to confirm the safety and efficacy of FMT. Future research and clinical trials should address this question. The effectiveness of CAR-T cell therapy for the treatment of MS also requires further clarification and confirmation. Overall, based on the available data, this approach could represent a new frontier in MS treatment and significantly advance the field in the near future. All the prerequisites are in place. However, the possibility of a safe clinical combination of CAR-T therapy and gut microbiota interventions remains unresolved because the gut microbiota may correlate with both treatment efficacy and neurotoxicity in CAR-T recipients. Gut microbiota modulation therapies, such as probiotics and FMT, are investigational and cannot be safely recommended for patients receiving treatment with CAR-T cells. Thorough, mechanism-rich studies are needed that combine gut microbiota monitoring with inflammatory marker measurements and neuroimaging before clinical application.

## Figures and Tables

**Table 1:** **Monoclonal antibodies currently approved or under clinical investigation for the treatment of multiple sclerosis (MS).** The table summarizes the mechanisms of action of each monoclonal antibody (mAb), their primary immune cell targets, FDA approval status, associated MS subtypes, and the corresponding phases and identifiers of representative clinical trials. Abs which are not FDA-approved are marked as N/A.

Agent	Mechanism of Action	Targeted Cells	Date of FDA Approval	Phase of Clinical Trials	Types of Multiple Sclerosis	Clinical Trials
Ocrelizumab	Humanized anti-CD20 mAb. Depletion of circulating mature and immature B cells by anti-CD20 dependent toxicity.	B cells	2017	III	RRMS	NCT02637856
				III	Relapsing MS	NCT02980042
				III	PPMS	NCT01194570
				IV	Relapsing MS	NCT03853746
				IV	Relapsing MS	NCT03589105
				IV	RRMS	NCT06495593
Rituximab	Chimeric anti-CD20 mAb. Similar to ocrelizumab.	B cells	N/A	III	RRMS	NCT05758831
				III	SPMS	NCT03315923
				III	PPMS	NCT00087529
				III	Relapsing MS	NCT04121403
				IV	RRMS	NCT03535298
Ublituximab-xiiy	Glycoengineered anti-CD20 mAb. Depletion of B cells via antibody-dependent cytotoxicity.	B cells	2022	III	Relapsing MS	NCT05877963
				III	Relapsing MS	NCT04130997
				III	Relapsing MS	NCT03277248
				III	Relapsing MS	NCT03277261
Ofatumumab	Human anti-CD20 mAb. Depletion of B cells via antibody-dependent cytotoxicity	B cells	2020	III	Relapsing MS	NCT04486716
				III	Relapsing MS	NCT04353492
				III	Relapsing MS	NCT02792231
				IV	Relapsing MS	NCT05199571
				IV	RRMS	NCT05084638
Frexalimab	Second generation anti-CD40L mAb. Blocking the costimulatory CD40/CD40L pathway in T- and B cells	T-cells B cells	N/A	II	Relapsing MS	NCT04879628
				III	Non-relapsing SPMS	NCT06141486
				III	Relapsing MS	NCT06141473
Natalizumab	Humanized mAb, which blocks the alpha4-beta1 integrin receptor on leucocytes	T-cells	2004 (revoked later)	III	RRMS	NCT03689972
				III	RRMS	NCT01440101
				III	SPMS	NCT01416181
				IV	RRMS	NCT00871780
				IV	RRMS	NCT02142205
				IV	RRMS	NCT03535298
Alemtuzumab	Humanized mAb, which targets CD52 on T- and B-lymphocytes and depletes these immune cells. Mechanisms of depletion include antibody and complement dependent cytotoxicity and apoptosis	T-cells (also can target B cells, macrophages, NK-cells and monocytes)	2014	III	RRMS	NCT00930553
				III	RRMS	NCT03477500
				IV	RRMS	NCT02255656
				IV	RRMS	NCT03135249
				IV	RRMS	NCT01395316
Daclizumab	Humanized mAb. It blocks CD25, the alpha unit of IL-2 receptor. Blockade of IL-2 inhibits T cell activation, expansion and survival	T-cells (also can target NK-cells)	2016	III	RRMS	NCT01064401
				III	RRMS	NCT01462318
Foralumab	Human anti-CD3 mAb. Suppression of T-cell proliferation and the release of proinflammatory cytokines	T-cells	N/A	II	Non-active SPMS	NCT06292923

**Table 2:** **Evidence-graded comparison of anti-CD20 monoclonal antibodies and anti-CD19 CAR-T cell therapy in multiple sclerosis.** The table highlights the key therapeutic characteristics, potential advantages, and current limitations of these two B cell-targeting strategies. Comparative features are classified as established, preliminary, preclinical or unknown. Established—supported by reproducible clinical data, large studies, and/or substantial longitudinal clinical use. Preliminary clinical evidence—supported by patient observations or early studies, but sample size, follow-up, and/or controls are limited. Preclinical evidence—supported only by in vitro or animal studies. Unknown/insufficient evidence—direct evidence is absent or insufficient for a reliable conclusion.

Comparative Feature	Anti-CD20 Monoclonal Antibodies	Anti-CD19 CAR-T Cells
MS-specific clinical efficacy	Established in multiple phase III trials and longitudinal clinical use.	Preliminary clinical evidence. There is limited evidence of efficacy from small studies.
Deep CNS parenchymal penetration	Unknown (insufficient evidence)	Preliminary evidence. Preliminary evidence on cell trafficking into cerebrospinal fluid, but deep penetration into CNS parenchyma requires confirmation.
Elimination of compartmentalized CNS B cells	Unknown (insufficient evidence)	Preclinical evidence; clinical evidence insufficient. Direct CNS B cell depletion was shown in cyclophosphamide-conditioned EAE. However, this finding has not yet been confirmed in patients.
Selective destruction of autoreactive B cells	Established. Broadly depletes CD20-expressing B cell subsets, but is not selective for autoreactive clones. CD20-negative plasmablasts and plasma cells are largely spared.	Established. Broadly targets CD19-expressing B-lineage cells, including many plasmablasts, but is not selective for autoreactive clones. CD19-negative long-lived plasma cells may be spared.
Treatment regimen	Established. Approved regimens use repeated maintenance dosing, and durable disease control is established on these schedules.	Preliminary data on the feasibility of a single infusion in MS therapy
Manufacturing and scalability	Established. Antibodies can be distributed through routine MS care pathways and are more scalable than autologous CAR-T therapy.	Established. Autologous therapy requires leukapheresis, individualized manufacturing and release testing, lymphodepletion, and specialist administration and monitoring.
Safety/side effects	Established. Large MS trials and post-approval use define risks, including infusion reactions, infections, hypogammaglobulinemia, nephropathy, thrombocytopenia, liver and brain damage. The frequency and severity vary depending on the agent and cumulative exposure.	Preliminary data report that patients had no significant side effects after the therapy. Possible risks derived mainly from oncology include CRS, ICANS, infections, prolonged cytopenia, hypogammaglobulinemia and LICATS.

## Data Availability

No new data were created or analyzed in this study. Data sharing is not applicable to this article.
